# The gut microbiome and metabolome in kidney transplant recipients with normal and moderately decreased kidney function

**DOI:** 10.1080/0886022X.2023.2228419

**Published:** 2023-06-29

**Authors:** Yang Lan, Duo Wang, Jiayang He, Hongji Yang, Yifu Hou, Wenjia Di, Hailian Wang, Xiangwei Luo, Liang Wei

**Affiliations:** a Clinical Immunology Translational Medicine Key Laboratory of Sichuan Province and Institute of Organ Transplantation, Sichuan Provincial People’s Hospital, School of Medicine, University of Electronic Science and Technology of China, Chengdu, China; b Chinese Academy of Sciences Sichuan Translational Medicine Research Hospital, Chengdu, China

**Keywords:** Chronic kidney disease after transplantation, kidney transplantation, gut microbiome, metabolites

## Abstract

**Background:**

The kidney transplant recipients (KTRs) were diagnosed with Chronic Kidney Disease after transplantation (CKD-T). CKD-T can be affected by the microbial composition and metabolites. The present study integrates the analysis of gut microbiome and metabolites to further identify the characteristics of CKD-T.

**Methods:**

We collected 100 fecal samples of KTRs and divided them into two groups according to the stage progression of CKD-T. Among them, 55 samples were analyzed by Hiseq sequencing, and 100 samples were used for non-targeted metabolomics analysis. The gut microbiome and metabolomics of KTRs were comprehensively characterized.

**Results:**

As well as significant differences in gut microbiome diversity between the CKD G1-2T group and CKD G3T group. Eight flora including Akkermansia were found to be enriched in CKD G3T group. As compared with CKD G1-2T group, the relative abundance of some amino acid metabolism, glycerophospholipid metabolism, amino acid biosynthesis, carbohydrate metabolism and purine metabolism in CKD G3T group were differential expressed significantly. In addition, fecal metabolome analysis indicated that CKD G3T group had a unique metabolite distribution characteristic. Two differentially expressed metabolites, N-acetylornithine and 5-deoxy-5'-(Methylthio) Adenosine, were highly correlated with serum creatinine, eGFR and cystatin C. The enrichment of gut microbial function in CKD-T is correlated with the expression of gut metabolites.

**Conclusion:**

Gut microbiome and metabolites in the progression of CKD-T display some unique distribution and expression characteristics. The composition of the gut microbiome and their metabolites appears to be different between patients with CKD G3T and those with CKD G1-2T.

## Introduction

Allogeneic kidney transplantation is the most effective treatment for kidney failure [1]. The short-term survival rates of kidney transplant recipients (KTRs) have been greatly increased with the development of surgical technology, but the long-term survival rate has undergone little improvement in recent years [2].

There are many factors that affect long‐term graft survival, such as rejection, metabolic disease, cardiovascular disease, infection and malignant tumors, and so on [3,4], and affect the survival quality of patients. At present, there is still a lack of personalized and comprehensive intervention system to maintain graft function.

The course of CKD-T (Chronic Kidney Disease after Transplantation) is inevitable after kidney transplantation. According to the Kidney Disease Improving Global Outcomes’s (KDIGO) diagnostic criteria: all KTRs should be considered to have Chronic Kidney Disease, irrespective of the GFR level or the presence of kidney injury markers of kidney damage [5]. When CKD develops into stage 3 and beyond, the kidney function of patients would no longer be able to sustain life for a long time [6]. The course of CKD-T may lead to many adverse outcomes, however, most clinical interventions for CKD-T prognosis focus on the control and prevention of complications., such as reducing the risk of cardiovascular diseases, screening for malignant tumors and controlling infectious complications. There is no intervention directly targeting the course and development of CKD-T [7]. So, finding novel diagnostic markers and therapeutic targets for CKD-T have great significance in preventing the progression of CKD-T.

Gut microecosystem is the biggest microecosystem in human being, which plays an important role in human health and diseases [8]. Specific changes of gut microbiome are associated with the development of CKD. A study by Ren et al. demonstrated that the changes in the proportion of Akkermansia and Butyricimonas were significantly associated with SCr and BUN and the development of CKD [9]. Another independent study found that Eggerthella lenta and Fusobacterium nucleatum increase uremic toxins production and promote kidney disease development in a CKD rat model [10]. Viewed the other way around, a progressive deterioration of renal function could also lead to changes in the intestinal flora [11]. Anders et al. believe that patients with chronic renal failure have impaired intestinal barrier function and affected the structure, composition, and function of gut microbiota due to the entry of urea and other residual toxins into the intestine [12]. However, few studies have investigated the characteristics changes of gut microbiome in the progression of CKD-T.

In this study, we aim to evaluate the population and function of gut microbiome using Hiseq sequencing and analyze fecal metabolic profiles using untargeted metabolomics method, to investigate the alterations of gut microbiome in CKD-T, and its effects on the related microbial functions and metabolites. We also identify the potential mechanism of CKD-T progression through analyzing the characteristics of gut microbiome and fecal metabolites in KTRs. These results may provide new strategies for individualized health management, prediction, diagnosis and treatment (such as probiotics and metabolites intervention, and flora transplantation) of CKD-T.

## Materials and methods

### Ethical approval

Our study has been approved by the Ethics Committee of Sichuan Provincial People’s Hospital (Ethical Approval No. 2021314) and was conducted in accordance with the Declaration of Helsinki. With written informed consent, all KTRs knew and approved the use of their clinical data and fecal samples for this study.

### Study design, participants recruitment and cohort grouping

**Figure 1.**
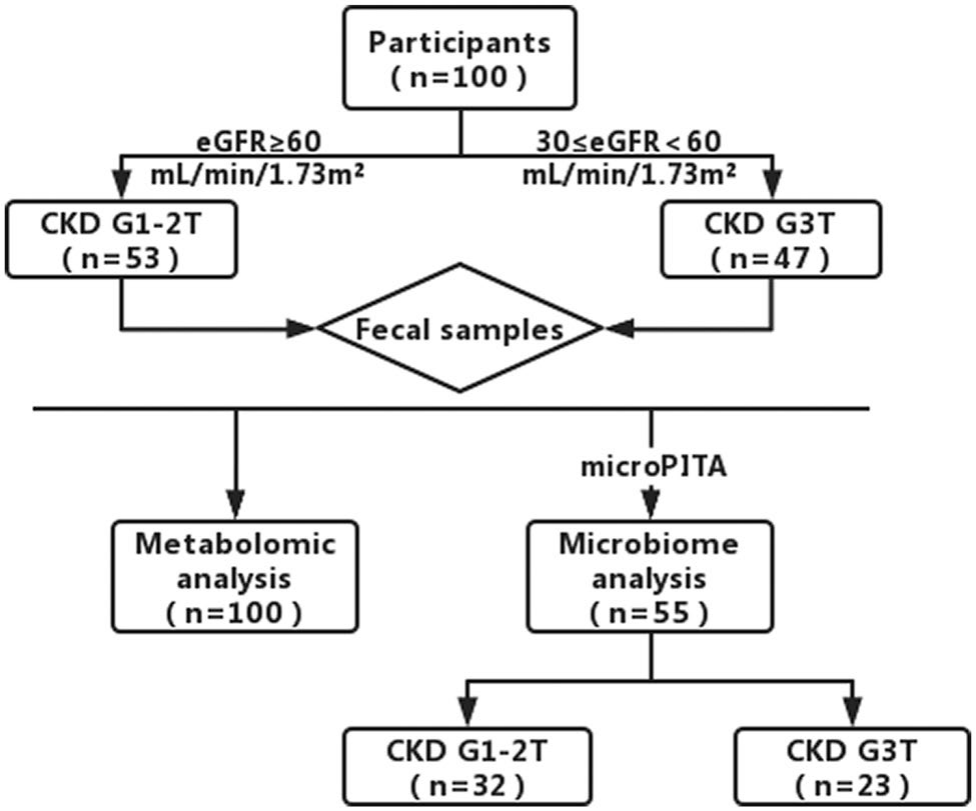
Trial design. A total of 100 KTRs fecal samples were collected for analysis, 100 of these fecal samples were used for metabolomics analysis and a total of 55 samples were selected for use metagenomic analysis.

As shown in Figure 1, a total of 100 KTRs with stage 1–3 were recruited to participate in this study, using the following criteria: (i) ≥3 months after kidney transplantation; (ii) stable graft function (serum creatinine concentration ≤ 2.0 mg/dL); (iii) immunosuppression regimen was stable in the last month. Recipients with kidney dysfunction or acute rejection which confirmed by biopsy within the past 3 months were excluded. A total of 100 KTRs fecal samples were collected for analysis, 100 of these fecal samples were used for metabolomics analysis, for metagenomic analysis, samples for sequencing were selected using microbiomes Picking Interesting Taxonomic Abundance (microPITA) to select typical samples of each group, a total of 55 samples were selected. According to Kidney Disease Improving Global Outcomes (KDIGO)’s diagnostic criteria for CKD-T [5], participants were divided into two groups based on estimated glomerular filtration rate (eGFR): CKD G1-2T group (eGFR ≥60 mL/min/1.73m^2^, *n* = 53) and CKD G3T group (30 ≤ eGFR <60 mL/min/1.73m^2^, *n* = 47).

### Fecal sample and data collection

All KTRs were given fecal sampling tube with detailed instructions for sample collection when they were having routine fasting blood tests. Fecal samples were collected using Sarstedt tubes (Sarstedt, Nümbrecht, Germany) filled with a preservative buffer. Collection of fecal samples (≥ 6 g) is completed on the day of CKD-T diagnosis and grading, transferred to laboratories within 2 h and stored at −80 °C until DNA extraction. When collecting feces, care was taken to avoid contamination by the environment and urine. Demographic information of the participants was recorded in Table 1, including age, sex, BMI, time since translation, kidney function parameters, immunosuppressive treatment, and life style. Moreover, the exposure assessment was conducted using questionnaire (Supplementary Material_1) with questions concerning several lifestyle variables, such as smoking, alcohol consumption, physical exercise and eating habits.

**Table 1.** General clinical characteristics of renal transplant recipients.

	CKD G1-2T (*n* = 53)	CKD G3T (*n* = 47)	*p*-Value
Sex (men/women)	40/13	36/11	0.897
Age, years	37.9 ± 10.9	42.7 ± 8.7	0.017
BMI	20.3 ± 2.6	21.5 ± 3.0	0.032
Time since transplantation, months	22.4 ± 18.5	25.9 ± 20.4	0.371
Renal function parameters			
Serum creatinine, µmol/L	95.7 ± 22.2	150.2 ± 38.6	<0.001
eGFR, mL/min	81.3 ± 15.4	48.5 ± 9.2	<0.001
Cystatin C, mg/L	1.5 ± 0.5	2.1 ± 0.6	<0.001
Urea, mmol/L	8.7 ± 10.1	10.4 ± 4.1	0.269
Immunosuppressive treatment			
Tacrolimus, *N* (%)	43(81.1)	36(76.6)	0.578
Blood concentration of tacrolimus, ng/ml	7.2 ± 2.8	6.5 ± 2.1	0.216
Cyclosporine, *N* (%)	10(18.9)	11(23.4)	0.578
Blood concentration of cyclosporine, ng/ml	93.6 ± 26.1	91.5 ± 32.1	0.874
Mycophenolate preparations (mycophenolate mofetil or mycophenolate sodium), *N* (%)	47(88.7)	38(80.9)	0.416
Life style			
Exercise	1.42 ± 2.84	2.19 ± 2.91	0.186
Staple food frequency (days)	6.73 ± 0.73	6.70 ± 0.72	0.868
Coarse grains	2.94 ± 2.10	2.62 ± 1.95	0.425
Fresh meat	5.44 ± 1.67	5.05 ± 1.97	0.286
Beans	1.27 ± 1.35	1.77 ± 1.57	0.095
Processed meat	0.59 ± 1.05	0.67 ± 0.75	0.683
Fresh fish	1.37 ± 1.36	1.23 ± 1.44	0.633
Milk	3.22 ± 2.44	3.99 ± 2.49	0.125
Fruit	3.14 ± 2.08	3.70 ± 2.39	0.214
Vegetables	5.31 ± 1.84	5.35 ± 2.00	0.918
Dietary supplements	0.79 ± 2.09	1.53 ± 2.64	0.122
Sweets	0.57 ± 0.97	0.56 ± 1.17	0.992
Non-alcoholic drinks	0.30 ± 1.05	0.39 ± 0.77	0.624
Alcoholic drinks	0.49 ± 1.32	0.24 ± 0.89	0.289

A *p*-value > 0.05 indicated that significant differences were not present, *p*-value < 0.05 indicated a difference, *p*-value < 0.01 indicated significant difference, and a *p*-value < 0.001 indicated an extremely significant difference.

### Untargeted metabolomics analytical strategy

Metabolite extraction. One hundred fecal samples of KTRs were subjected to Novogene Co., Ltd (Beijing, China) for untargeted metabolomics analysis based on the liquid chromatography-mass spectrometry (LC-MS) method. The fecal samples were ground in liquid nitrogen, and 80% methanol aqueous solution containing 0.1% formic acid was added. After vortexing, samples were incubated on ice for 5 min, and centrifuged at 15,000 rpm for 10 min at 4 °C. Then, the supernatant was collected and diluted with LC-MS grade water until the methanol content is 53%. After that, a second centrifugation at 15,000 g for 10 min at 4 °C was performed, the supernatant was collected and used for LC-MS analysis. A mixed sample which was equally taken from each sample was used for quality control. And a 53% methanol aqueous solution containing 0.1% formic acid was used as the blank sample.

UHPLC-MS/MS analysis. UHPLC-MS/MS analyses were performed using a Vanquish UHPLC system (ThermoFisher, Germany) coupled with an Orbitrap Q Exactive™ HF mass spectrometer (Thermo Fisher, Germany). According to the requirements of the test standard, the system was equipped with Hypesil Goldcolumn(C18) (100 × 2.1 mm, 1.9 μm). The flow rate was set to 0.2 mL per minute. The eluents for the positive polarity mode were eluent A (0.1% formic acid in water) and eluent B (Methanol). The eluents for the negative polarity mode were eluent A (5-mM ammonium acetate, pH 9.0) and eluent B (methanol). The solvent gradient was set as follows: 2% B, 1.5 min; 2–100% B, 12 min; 100% B, 14 min; 100–2% B, 14.1 min; 2% B, 17 min. Column temperature was kept at 40 °C. The ESI source was set in the full-scan mode with a scan range of *m/z* 100–1500. The parameters included spray voltage of 3.2 kV, sheath gas flow rate of 40arb, Aux Gasflow rate of 10arb, capillary temp of 320 °C and MS/MS secondary scans were data-dependent.

Data processing and metabolite identification. The LC-MS data analysis raw data files generated by UHPLC-MS/MS were processed using the Compound Discoverer 3.1 (CD3.1, ThermoFisher) for peak alignment, peak picking and quantitation. The major parameters include retention time tolerance, 0.2 min; actual mass tolerance, 5 ppm; signal intensity tolerance, 30%; signal/noise ratio, 3; and minimum intensity,100,000. Afterward, peak intensities were normalized using the total intensity for each spectrum. the molecular formula was predicted based on molecular ion peaks and fragment ions, and compared with mzCloud (https://www.mzcloud.org/), mzVault and MassList database. At the same time, blank samples were set up to remove background ions. Statistical analyses were performed using the statistical software R (R version R-3.4.3), Python (Python 2.7.6 version), and CentOS (CentOS release 6.6). When data were not normally distributed, normal transformations were attempted using of area normalization method [13].

### DNA extraction, metagenomic sequencing and sequence data process

DNA extraction. The total genomic DNA in each fecal sample was extracted using the Hexadecyl trimethyl ammonium bromide (CTAB) method [14]. According to the manufacturer’s instructions, fecal DNA was isolated using the Tiangen Stool DNA extraction magnetic bead kit (Tiangen, Beijing, China). The main reagents used include phenol (pH 8.0), chloroform, and isoamyl alcohol (25:24:1), chloroform and isoamyl alcohol (24:1), and isopropanol. The quality of DNA was determined through 1% agarose gel electrophoresis. DNA was quantitated using the Qubit.

Construction of DNA Library. The DNA library was constructed according to the Novogene Co., Ltd (Beijing, China) manufacturer’s instructions. NEBNext Ultra™ DNA Library Prep Kit for Illumina (New England Biolabs, Ipswich, MA, USA) was used to generate sequencing libraries. Index codes were added to each sample to identify attributes for each sequence. The qualified DNA samples were randomly sheared to a size of 350 bp by sonication. Then these fragments were end-repaired, ligated with Illumina sequencing adapters, and PCR amplified. AMPure XP system (Beckman Coulter, CA, USA) was used to purify the PCR products, Agilent 2100 Bioanalyzer (Agilent Technologies, Santa Clara, CA, USA) was used to analyze the size distribution of DNA library and real-time PCR was used to quantify DNA libraries [15].

Sequencing and Data Preprocessing. Sequencing and processing were performed on an Illumina HiSeq platform by Novogene Co., Ltd (Beijing, China).

According to the manufacturer’s protocol, the clustering of index-coded samples was performed on a cBot Cluster Generation System (Illumina, San Diego, CA, USA) and grouping according to operational taxonomic units (OTUs) were performed using the UPARSE pipeline with 97% consistency. The library preparations were then sequenced on an Illumina Hiseq platform and paired-end reads generated. Raw reads can be processed according to three filtering standards. (a) Reads with low-quality bases were removed (quality value ≤ 38; length was set up to 40 bp). (b) The reads containing N base that reached a certain percentage were removed (length was set up to 10 bp). (c) The reads which shared the overlap above 15 bp with adapter were removed. Preprocessing the Raw Data obtained from the Illumina HiSeq sequencing platform using Readfq (V8, https://github.com/cjfields/readfq) was conducted. And the clean reads were assembled using the MEGAHIT (version 1.0.4-beta) with default parameters. Metagenemark (V2.10, http://topaz.gatech.edu/GeneMark/) was used to predict the open reading frames (ORFs) of samples and scaftigs (≥ 500 bp) [16], and fragments < 100 nt were filtered out. All samples’ clean data are compared to each Scaffolds respectively by Bowtie2 (Bowtie2.2.4) software to acquire the PE reads not used and the parameters. Filter the gene which the number of reads < 2 [17–18] in eachsample and statistic the abundance information of each gene in each sample. DIAMOND (Integrative analysis of environmental sequences using MEGAN4) software (V0.9.9, https://github.com/bbuchfink/diamond/) is used to blast the Unigenes to the sequences of Bacteria, Fungi, Archaea and Viruses which are all extracted from the NR database (Version: 2018-01-02, https://www.ncbi.nlm.nih.gov/) of NCBI.

### Statistical analysis

Analysis of the Microbiota. Krona analysis, the exhibition of generation situation of relative abundance, the exhibition of abundance cluster heat map, principal coordinate analysis (PCoA) [19] (R ade4 package, Version 2.15.3) and non-metric multidimensional scaling (NMDS) [20] (R vegan. package, Version 2.15.3) decrease-dimension analysis are based on the abundance table of each taxonomic hierarchy. The difference between groups is tested by Anosim analysis. Metastats and LEfSe analysis are used to look for the different species between groups. LEfSe analysis is conducted by LEfSe software (the default LDA score is 3) [21]. Random forest (RandoForest) (R pROC and randomForest packages, Version 2.15.3) was used to construct a random forest model. A heat map reveals the function difference between microbial communities of CKD G1-2T and CKD G-3T. (Zscore method is applied to the preprocessing of original data.). Screen out important species by MeanDecreaseAccuracy and MeanDecreaseGin, 24 CKD G1-2T and 22 CKD G3T samples were randomly divided into the training set, and the remaining participants were incorporated into the testing set. Then cross-validate each model (default 10 times) and plot the ROC curve. Adopt DIAMOND software (V0.9.9) to blast Unigenes to functional database. Statistic of the relative abundance of different functional hierarchy.

Metabolomic Analysis. The KEGG database (https://www.genome.jp/kegg/pathway.html), HMDB (https://hmdb.ca/metabolites) and LIPIDMaps database (http://www.lipidmaps.org/) were used to annotate the identified metabolites. Principal components analysis (PCA) and Partial least squares discriminant analysis (PLS-DA) were performed at metaX. One-way ANOVA analysis was used for comparison of baseline characteristics between different groups. We applied univariate analysis (t-test) to calculate the statistical significance (*p*-value), and the fold change (FC value) of the metabolites between the two groups was calculated. When the variable importance for the projection (VIP) values > 1 and *p*-value < 0.05 and fold change ≥2 or FC ≤ 0.5, significantly different metabolites were identified. For clustering heat maps, the data were normalized using z-scores of the intensity areas of differential metabolites and were ploted by Pheatmap package in R language. The correlation between differential metabolites were analyzed using cor function in R language (method = pearson). The R function cor.mtest was used for significance tests and correlation matrix visualization. *p*-value < 0.05 was considered as statistically significant and correlation plots were ploted by corrplot package in R language. The functions of differential metabolites and metabolic pathways were studied using the KEGG database. The metabolic pathways enrichment of differential metabolites was performed, when ratio were satisfied by x/n > y/N, metabolic pathway were considered asenrichment, when *p*-value of metabolic pathway < 0.05, metabolic pathway were considered as statistically significant enrichment.

Statistical analyses were performed using the SPSS V.25.0 and Graphpad Prism V.8.0. Numerical data are presented as mean ± standard deviation. One-way ANOVA analysis was used for the comparison of baseline characteristics between different groups. Wilcoxon rank-sum test was used to compare the differences between two independent groups. *p*-Value < 0.05 was considered statistically significant.

## Results

### Clinical characteristics of KTRs

As shown in Table 1, no statistical differences were found in gender, body mass index (BMI), time since transplantation and life styles between the two groups. While the difference in age, creatinine, eGFR and cystatin C between the two groups were statistically significant. Moreover, there were also no significant differences in blood concentrations of tacrolimus and cyclosporine A between these groups.

### Gut microbial diversity of patients with CKD G3T was increased

**Figure 2.**
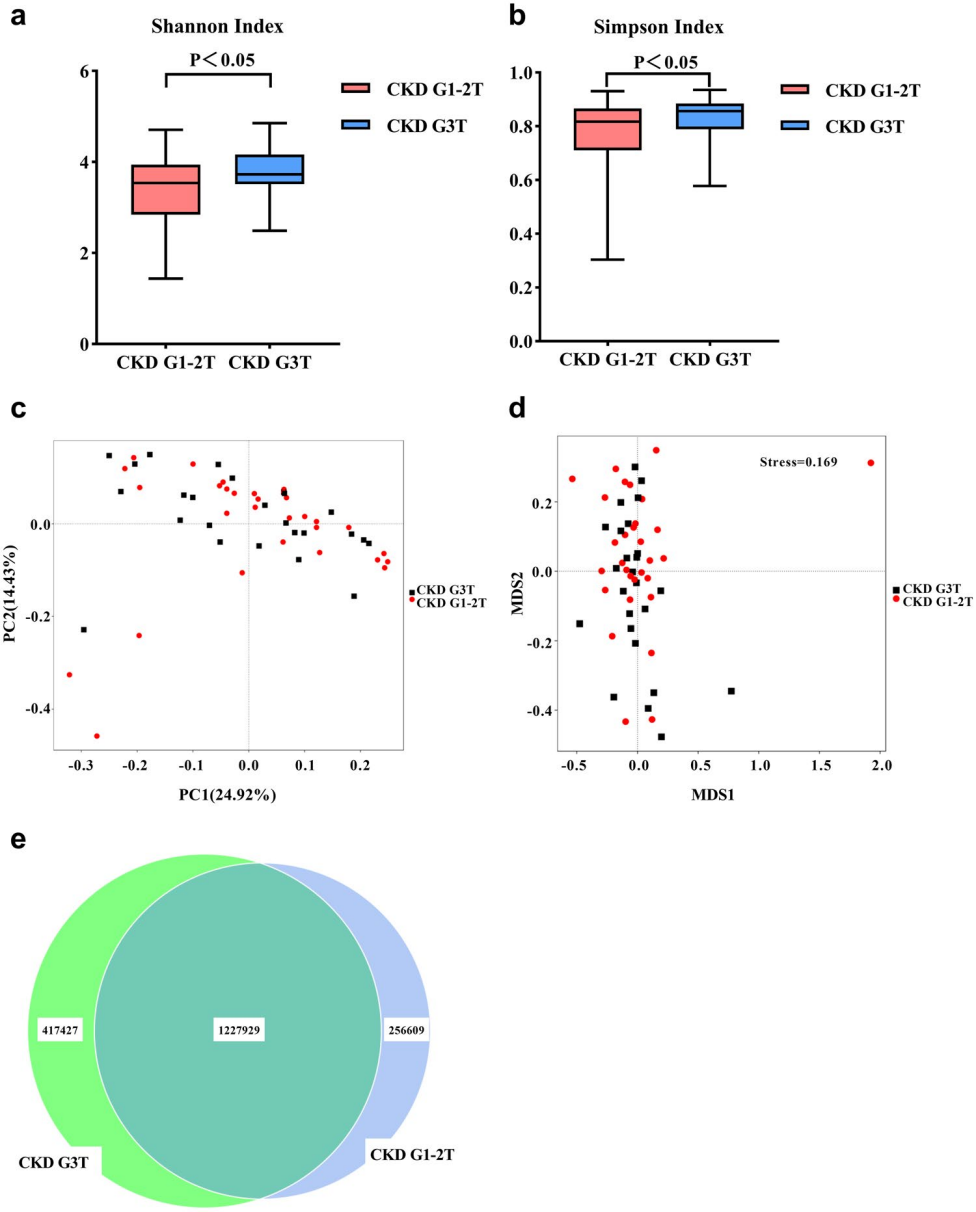
Gut microbial diversity between CKD G1-2T and CKD G3T groups. α-diversity analysis: (a) shannon index; (b) simpson index. β-diversity analysis: (c) principal coordinates analysis (PCoA) (based on Bray-Curtis distance); (d) nonmetric multidimensional scaling (NMDS). (e) Venn diagram showing the overlap of OTUs.

Our study found a statistically significant difference in gut microbial diversity between the two groups, which was estimated by Shannon index and Simpson index (Figure 2(a,b)). To evaluate the microbiome space between different groups, the β diversity was calculated by weighted Unifrac, the results of principal coordinate analysis (PCoA) and the non-metric multidimensional scaling (NMDS) analysis showed that there were no statistically significant differences in the composition of gut microbiome between the two groups (Figure 2(c,d)). Fewer total OTUs in the CKD G1-2T group than in the CKD G3T group were observed in the Venn diagram: 417,427 of 1,645,356 OTUs were unique to CKD G3T, and 256,609 of 1,484,538 OTUs were unique to CKD G1-2T (Figure 2(e)).

### Phylogenetic profiles of gut microbial in CKD-T

**Figure 3.**
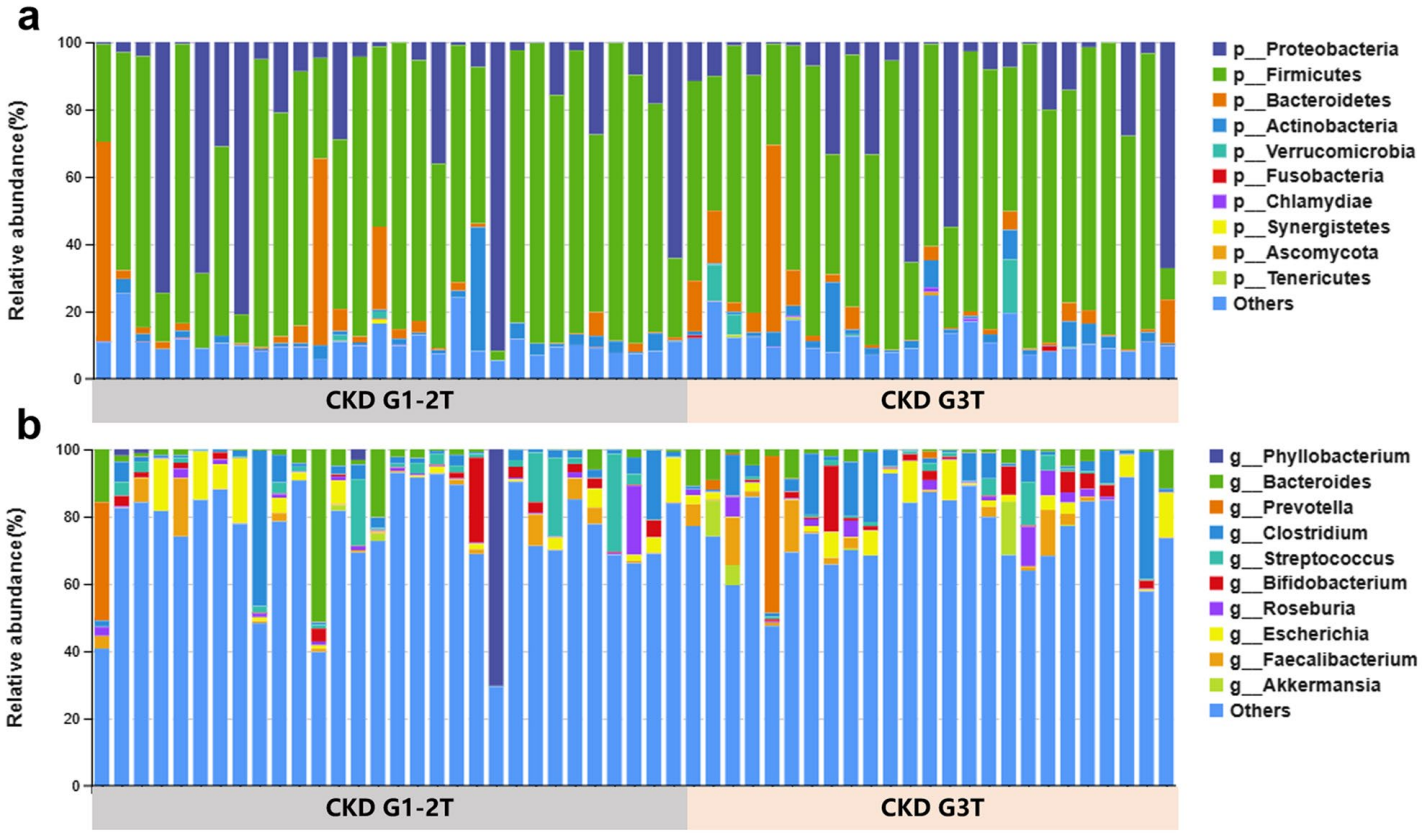
Phylogenetic profiles of gut microbes in CKD G1-2T and CKD G3T groups. Fecal microbial composition of the CKD G1-2T and CKD G3T groups at the phylum and genus levels (a,b).

To evaluate the differences in flora composition, the relative abundance levels of the phylum and genus with the top 10 total abundances were analyzed in different groups. As shown in Figure 3(a), three of the phyla, Firmicutes, Proteobacteria and Bacteroidetes accounted for more than 80% of the sequences on average, which could be regarded as the dominant species in KTRs. With the decrease of eGFR, the relative abundance of Firmicutes increased, while Proteobacteria and Bacteroidetes decreased, at the phylum level. Meanwhile, at the genus level, the relative abundance of Prevotella and Clostridium genus increased and Bacteroides and Escherichia genus decreased (Figure 3(b)).

### Major bacteria and microbial function related to CKD G3T

**Figure 4.**
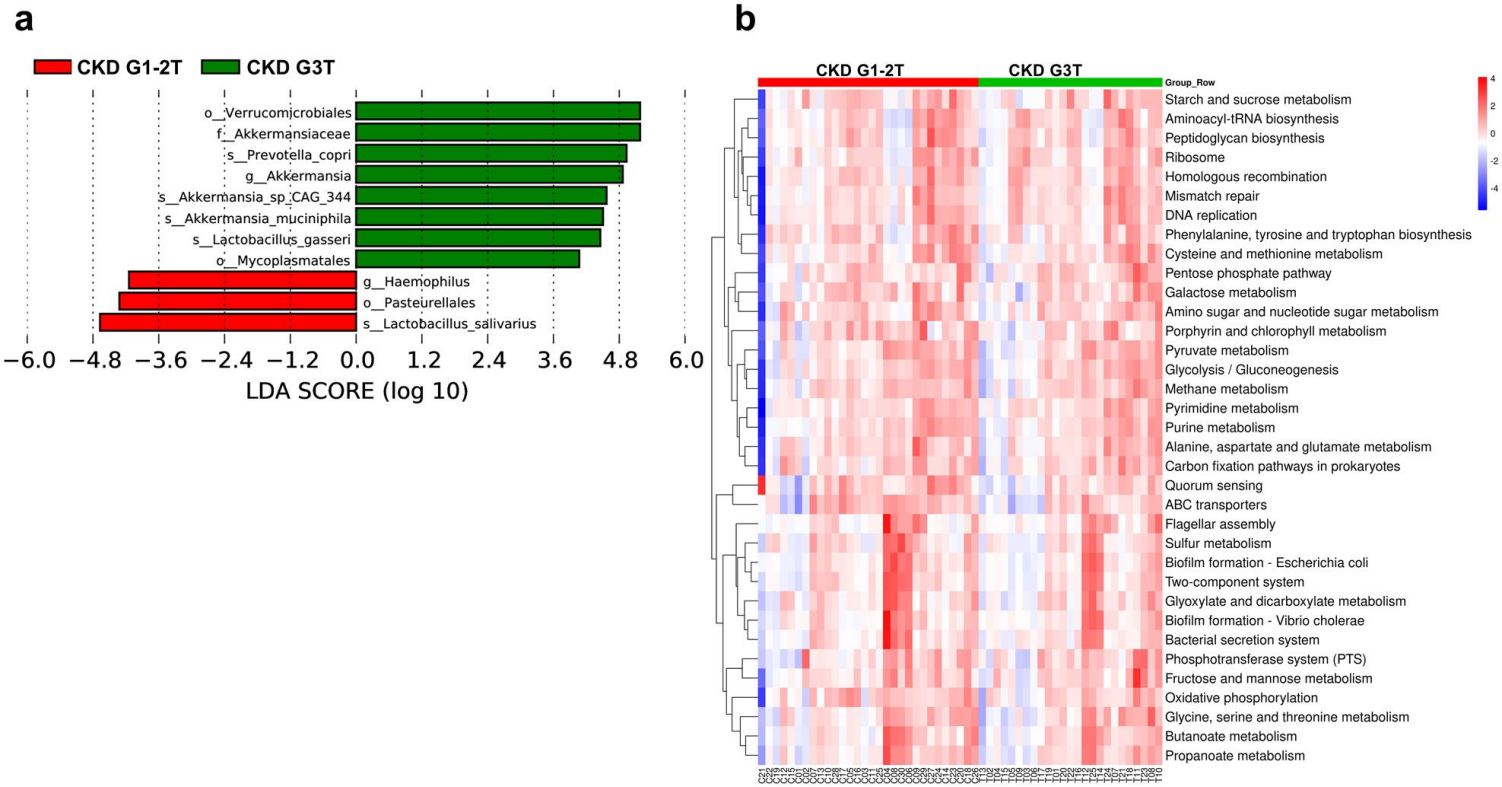
LEfSe analysis and predicted function in the gut Micorbiota of CKD G1-2T and CKD G3T groups. (a) LDA score histogram of CKD G1-2T and CKD G3T groups. (b) Heatmap of microbial function pathways.

Linear discriminant analysis effect size (LEfSe) was used to estimate the maximum difference of the microbial structures between these two groups, to determine the specific bacterial taxa and predominant bacteria which were associated with CKD G3T. Our LEfSe analysis based on LDA > 4 found that 11 taxa (phylum to genus) in CKD G3T group and CKD G1-2T group significantly enriched (Figure 4(a)). PICRUSt revealed the taxonomic distribution of metabolic functions between CKD G1-2T and CKD G3T.

To further explore the biological metabolic pathways, the functional annotation analysis was performed through searching and comparing the genomic data generated by metagenomic sequencing in the Kyoto Encyclopedia of Genes and Genomes (KEGG), and a heatmap was generated based on annotations. As shown in Figure 4(b), the functions of Amino acid (cystine and methionine) metabolism, glycerol phospholipid metabolism, fructose and mannose metabolism and purine metabolism were significantly increased, while the metabolism of amino acids (e.g., glutamic acid and threonine), biosynthesis of amino acids (e.g., tyrosine and tryptophan), carbohydrates metabolism (e.g., starch and sucrose, lactose), and propionic acid and ketone propionate metabolism were significantly decreased, when compared with the CKD G1-2T group.

### Diagnostic potential of CKD G3T based on the gut microbial markers

**Figure 5.**
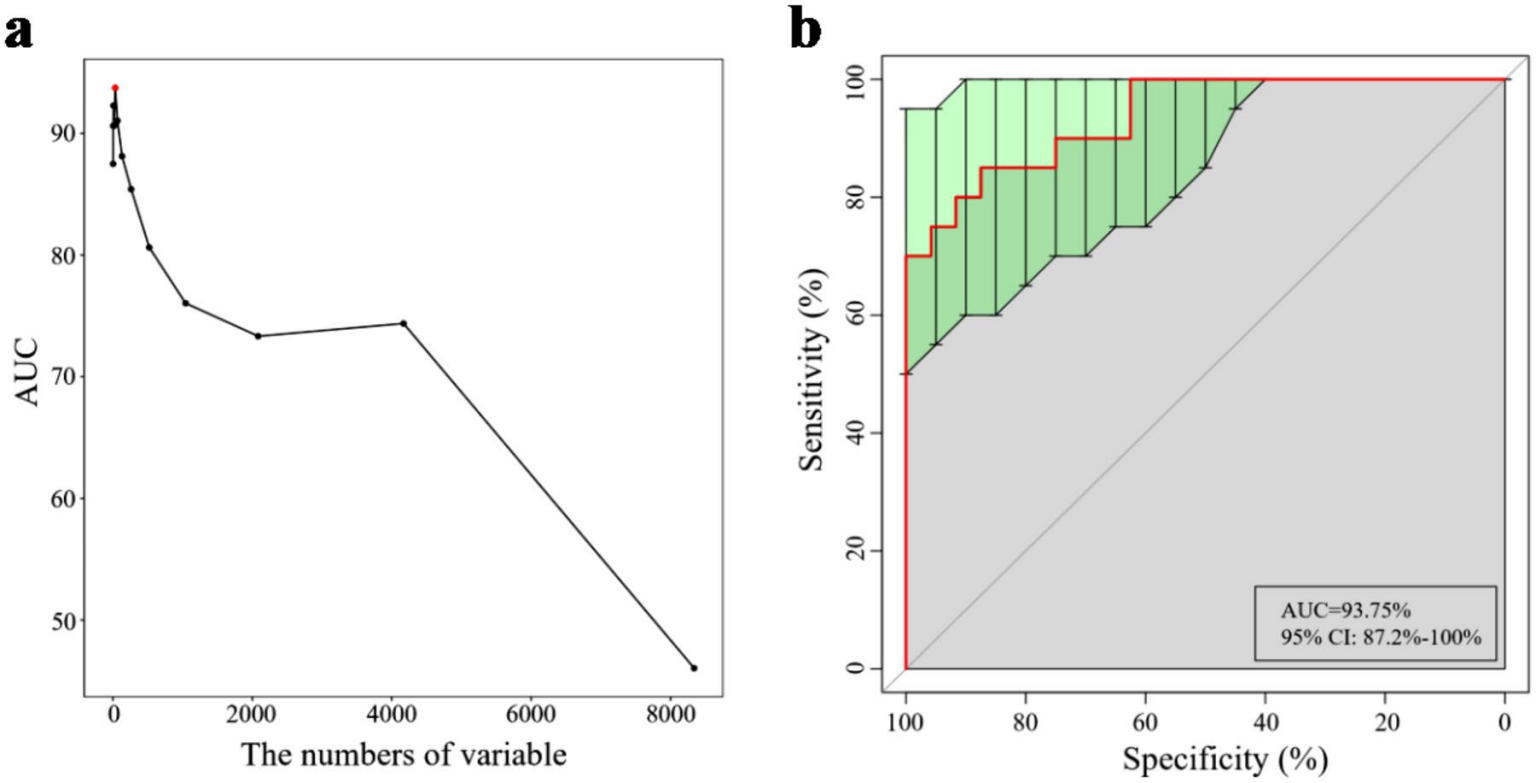
The importance of fecal microbiota to CKD G3T assessed using the random forest model. (a) a total of 33 OTU markers can be used as the optimal marker set. (b) The POD index achieved an AUC value of 93.75% (95% CI: 87.2%–100%).

We performed a tenfold cross validation on the random forest model of the two groups, to identify unique OTU markers of the CKD G3T group. The result showed that there were 33 OTU markers to be selected as the best marker set (Figure 5(a)). The POD index achieved an AUC value of 93.75% with 95% CI of 87.2% to 100% between the two groups (Figure 5(b)). The data indicated that the classifier model based on microbial markers reached a powerful diagnostic potential in distinguishing CKD G3T.

### Differences in metabolite profile

**Figure 6.**
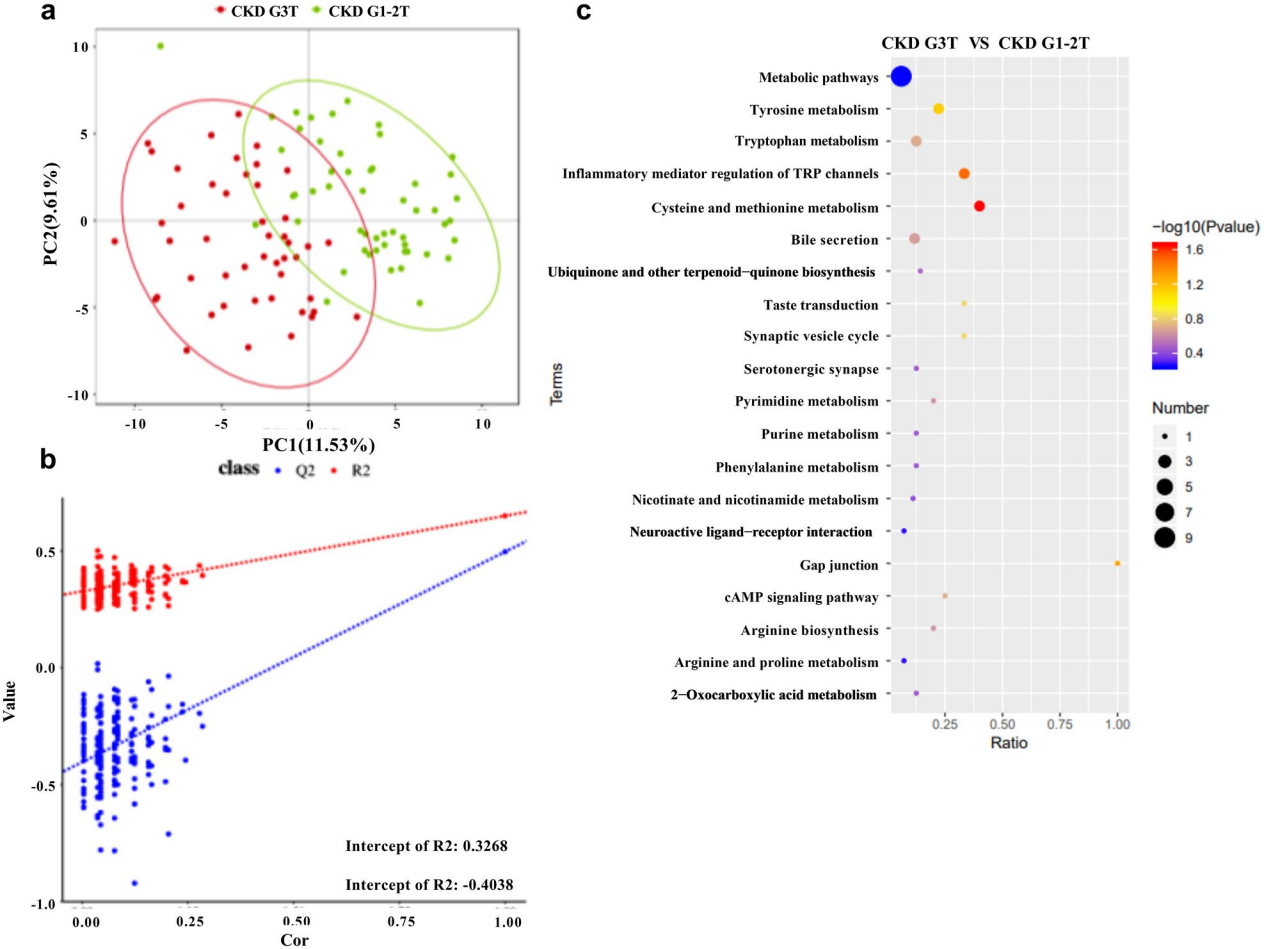
Metabolomics analysis of fecal samples. (a,b) PLS-DA score plots and permutation test of CKD G1-2T and CKD G3T group. (c) KEGG pathway analysis of differentially expressed metabolites.

We conducted fecal metabolic profiling in the 100 study subjects by LC-MS/MS, using positive ion modes. To identify the differential metabolites between two groups, partial least squares discriminant analysis (PLS-DA) was used to build a model which could evaluate the correlation between metabolite expression level and KTR status. The PLS-DA model demonstrated significant clustering of samples from the 2 groups (R^2^Y = 0.71, Q^2^ = 0.49) (Figure 6(a,b)).

Based on the PLS-DA models, differentially expressed metabolites between groups were identified using the following criteria: variable importance in projection (VIP) values > 1.0, fold change of mean expression level between groups > 1.2 or < 0.833, and *p*-value < 0.05. A total of 52 differentially expressed metabolites were identified between the CKD G1-2T and CKD G3T groups, of which 20 metabolites were significantly decreased and 32 metabolites were significantly increased in the CKD G3T group (Supplementary material_2).

KEGG analysis revealed the main pathway of metabolite enrichment in differential expression (Figure 6(c)). Based on the results of our KEGG pathway analysis of gut microbiota and fecal metabolomics, as well as published reports, we focused on amino acid metabolism and biosynthesis. N-Acetylornithine, Serotonin, D-Cysteine, 5-Methoxytryptamine, Hydroxyproline, 5′-Deoxy-5′-(Methylthio) Adenosine were significantly decreased, while Capsaicin, Rosmarinic acid and Homogentisic Acid were significantly increased in CKD G3T group.

### Correlation of metabolites with kidney function

**Figure 7.**
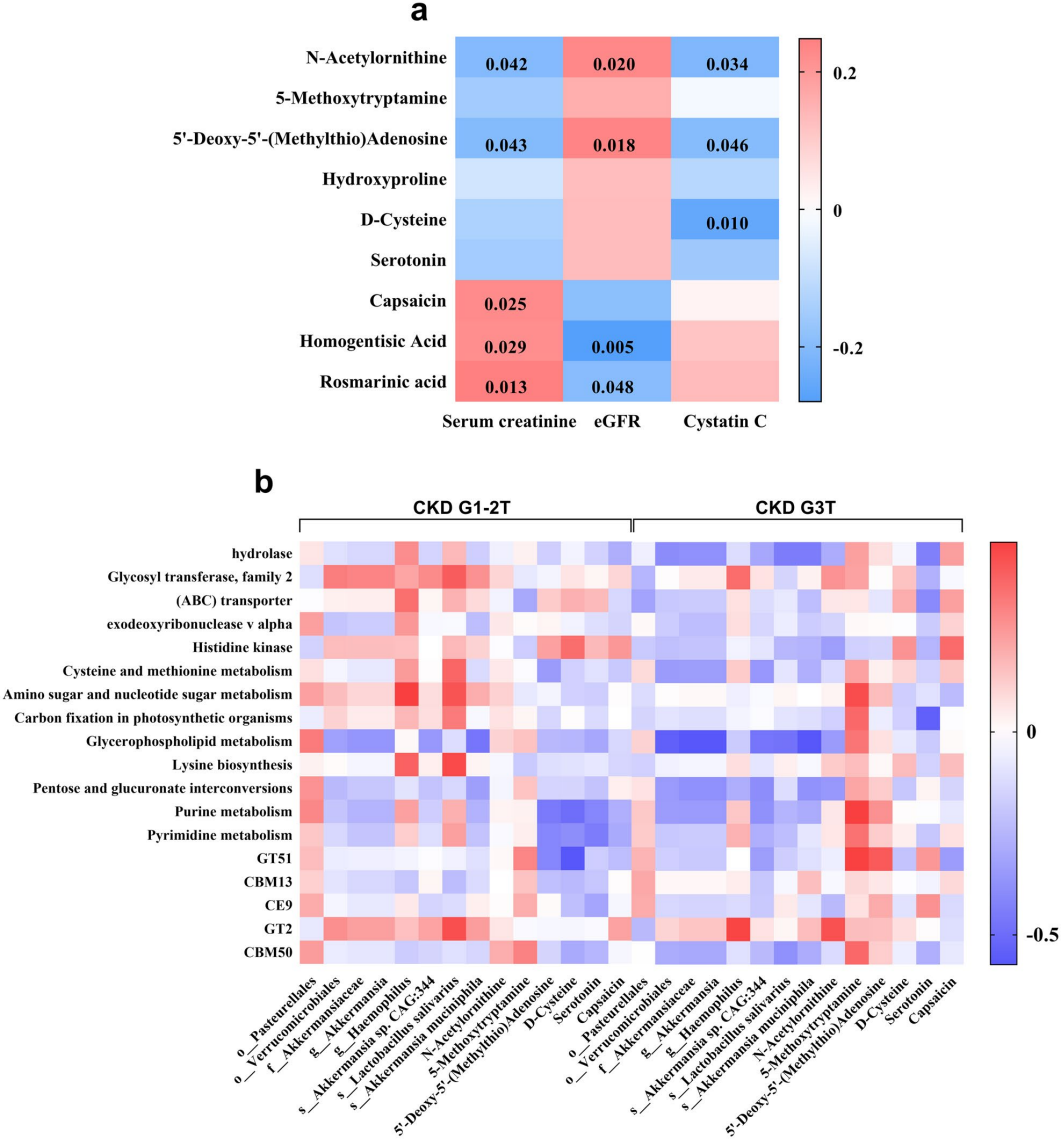
The characteristics of microbiota and its metabolites. (a) Correlational analysis of metabolites and kidney function indexes. (b) Analysis of correlations between key microbes and microbial functions in CKD G1-2T and CKD G3T groups. *p* < 0.05 indicated a difference, *p* < 0.01 indicated significant difference.

The correlation of different metabolites with kidney function was evaluated by calculating Spearman’s rank correlation coefficient. As shown in Figure 7(a), only the expressions of N-Acetylornithine and 5′-Deoxy-5′-(Methylthio)Adenosine were correlated with eGFR, Cystatin C and serum creatinine (*p* < 0.05).

### Correlation among differentially expressed metabolites, gut microbiome and microbial function

To further explore the correlation between the diversity of gut microbiome and representative differentially expressed metabolites in CKD G3T, we analyzed the abundance of the major gut microbiome and fecal metabolites. As shown in Figure 7(b), many microbial functions were significantly covariant with the concentrations of gut microbiome and metabolites in the CKD G3T group, when compared with the CKD G1-2T group.

## Discussion

The role of gut microbiota in CKD were gradually explored [9], but so far, few studies have investigated the role of gut microbiota on the development of CKD-T. In this report, the gut microbiome and metabolome of fecal samples from patients with CKD-T were fully characterized with metagenomics sequencing and untargeted metabolomics analysis, and underlines the gut microbiome and metabolome may further change in the course of CKD-T.

Compared to the CKD G1-2T group, there was an obvious increase in the relative abundance of gut microbiota in the CKD G3T group (*p* < 0.05). Weighted UniFrac distances were used to evaluate β-diversity and found that the gut microbiota composition between the two groups were similar. We speculated that there may be two reasons. First of all, due to immunosuppressive drugs used by KTRs may induced significant changes in the structure of intestinal microflora [11], there were no significant difference in β-diversity was observed between the two groups. And secondly, while α-diversity reflects the richness of gut microbiota within the populations, which is different from measuring the composition and abundance of the gut microbiome among the populations. There were significant differences in the α-diversity between the two groups, suggested that differences in the intestinal microflora between GFR categories could be the critical factors to explain the difference. Then, uric acid is the final product of purine metabolism in humans [22], and is normally excreted out of the body *via* the kidneys in the urine. In the case of CKD, it is instead excreted by the colon, along with oxalate [23]. In this process, the urease produced by intestinal bacteria can decompose this urea into ammonia, leading to changes in luminal pH [24]. That’s associated with alterations of gut microbiota. On further analysis, the gut microbiomes of KTRs at the phylum and genus levels were compared. We observed higher relative abundances of *Firmicutes*, *Prevotella*, *Clostridium*, *Faecalibacterium*, *Akkermansia*, etc., in patients with CKD G3T. According to LEfSe analysis, 11 taxa (phylum to genus) in CKD G3T group and CKD G1-2T group significantly enriched, eight taxa were abundant in CKD G3T group, these include *Verrucomicrobiales*, *Mycoplasmatales*, *Akkermansiaceae*, *Akkermansia*, *Prevotella copri*, *Akkermansia Sp: CAG_344*, *Akkermansia muciniphila* and *Lactobacillus gasseri*. This further proved that the composition of gut microbiota in patients with CKD G3T was changed. This means that, a progressive deterioration of renal function may lead to changes in these gut microbiota. Ren et al. [9] reported that the abundance of *Akkermansia* increases with the development of CKD. The abundance of *Akkermansia* had a positive correlation with the level of SCr and BUN, and had a negative correlation with the level of eGFR and hemoglobin. At the family and genus level, the Akkermansiaceae were significantly enriched in CKD G3T group in this study. This is the same trend as described in previous studies research on the correlation between gut mycobiota and CKD. This finding implies that it plays an important role in the reach of kidney function after transplantation. The mechanism of action deserves further excavation and investigation. Since CKD and CKD-T occur with eGFR < 60 mL/min/1.73m^2^ and decline in kidney function, they possess similar pathological characteristics [8]. However, we found that the gut microflora associated with the progression of CKD-T were not entirely consistent with CKD [25], such as changes in *Prevotella copri*. Unique changes in intestinal microbiota due to CKD-T progression are based on two potential explanations. First, it is reported that the balance of intestinal microbiota in KTRs is disturbed compared with non transplant healthy people [26]. Secondly, in addition to the traditional risk factors related to CKD, the development of CKD-T is also affected by three factors (adverse effects of immunosuppressive drugs, viral infection after transplantation and hypomagnesaemia) [27].

Furthermore, microbial function is also related to the development of disease. In this study, the function of cystine and methionine metabolism, fructose and mannose metabolism, purine metabolism etc were significantly increased in patients with CKD G3T. However, the function of amino acid metabolism(such as alanine and threonine), amino acid biosynthetic (such as tryptophan and tyrosine), carbohydrate metabolism(such as starch, sucrose and lactose), propanoate and pyruvate metabolism etc. were significantly reduced. Uric acid is the final metabolite of purine metabolism and is an independent risk factor in kidney failure. The disorder of purine metabolism leads to abnormality in uric acid. Tryptophan biosynthesis can increase the possibility of indole production. Phenylalanine biosynthesis contributes to the production of p-cresol [28]. The gut microbiota decomposes tyrosine and tryptophan to produce phenols (p-cresol and phenol) and indole, forming a uremic toxin – indole sulfate. Theoretically, the content of indole in the stool of patients at the end of CKD is higher. In patients with ESKD, fecal indole tends to be higher in the patients with CKD as compared to the healthy controls. We hypothesized that indole-producing metabolic pathways are more active in patients with CKD G3T. However, we found that the amino acid metabolic function of indole production in CKD G3T group is lower than that in CKD G1-2T group. A study on the gut microbiota of patients with CKD showed that there was no significant difference in the gut microbiota of indole production between the control group and the CKD group, and it was believed that the difference in the content of indole in feces was caused by decreased excretion, but this result still needs further confirmation [29]. In addition, our study found that the intestinal carbohydrate metabolism and propanoate and pyruvate metabolism of CKD-T 3 patients were significantly reduced. So far, there are no more studies confirming the correlation between these functional changes and CKD-T. Their significant changes in the gut microbiota of patients with CKD G3T suggest that they may have some physiological significance in the occurrence and development of CKD-T. This is a direction worth further research.

Meanwhile, a non-targeted metabolomic analysis was conducted on fecal samples collected from 100 KTRs to gain insight into the alterations in their metabolomic profiles. The PLS-DA results revealed a clear separation between the two groups of recipients, it suggests that fecal metabolites can be used as molecular markers for early screening of chronic kidney failure after kidney transplantation. A total of 85 metabolites were differentially expressed in the CKD G3T patients as compared to the CKD G1-2T patients. The 85 differential metabolites were analyzed by KEGG annotation to find pathways involved in regulating these differential metabolites. Because aromatic amino acids is significantly associated with CKD progression, our study focuses on changes in amino acid-related metabolic pathways.

The pathways involved in cysteine and methionine metabolism, tyrosine metabolism, arginine biosynthesis, phenylalanine metabolism, arginine and proline metabolism, and tryptophan metabolism are all related to the synthesis and breakdown of amino acids. Further analysis of the data revealed the expression of N-Acetylornithine and 5′-Deoxy-5′-(Methylthio) Adenosine was significantly correlated with kidney function-related indexes (serum creatinine, eGFR and cystatin C). Circulation levels of five different N-acetylated amino acids are associated with kidney failure. According to Wang et al. [30], N-acetylated amino acids is positively correlated with arginine metabolism, and negative correlation with lysine and anthranilate. The upregulation of N-Acetylornithine also correlates with kidney development. There has been a study indicating that 5′-Deoxy-5′-(Methylthio) Adenosine is associated with acute kidney injury (AKI) in patients with type 2 diabetes mellitus (T2DM) undergoing off-pump coronary artery bypass grafting (CABG) [31]. Although there is no research showing the correlation between the change of 5′-Deoxy-5′-(Methylthio) Adenosine and CKD-T. However, our data shows that the significant changes of 5′-Deoxy-5′-(Methylthio) Adenosine suggesting that it has important physiological significance in the course of CKD-T. This is the direction that we need to continue to study in the future.

As a buffer for host perception and response to internal and external environmental disturbance, gut microbiota plays a vital role in many aspects of human health, including immunity and metabolism, and can interact with different basic biological processes of the host through the production and release of metabolites [32–34]. Multi-omics studies will accelerate our understanding of the contribution of gut microbiota to human health and metabolic diseases [35] and may improve global awareness of changes in the function of chronic kidney disease after kidney transplantation. The mechanisms of the key intestinal flora and metabolites identified in this study in the progression of CKD-T need further clarification.

The study has certain limitations. We only analyzed one stool sample per participant, due to the limitation of the sample source. We were unable to clarify the causal relationship between the microbiome and the development of CKD-T. We can only prove that there is a connection between them. It is worth noting that due to the complexity of pharmacokinetics and inter-patient variability, large differences in methods of immunotherapy were display between individuals. Although we found no significant difference in the blood drug concentration of the main immunosuppressive agents between the two groups, this is not sufficient to completely exclude the impact of immunosuppressive agents on microorganisms. Otherwise, there is a relative dearth of studies that examine CKD-T. It is necessary to conduct a large prospective cohort investigation and analyses of other potentially significant variables in order to confirm the associations observed in the current study.

## Conclusion

In conclusion, gut microbiome and metabolites in the progression of CKD-T display some unique distribution and expression characteristics. And strong links between CKD-T and gut microbial dysbiosis suggest potential therapeutic strategies to prevent CKD-T progression.

## Supplementary Material

Supplemental Material

Supplemental Material

## Data Availability

Source data for information presented in this study are available from the corresponding authors on reasonable request.
