# Supplementary material for: The gut microbiome and metabolome in kidney transplant recipients with normal and moderately decreased kidney function
Source: Ren Fail. 2023 Jun 29;45(1):2228419. doi: 10.1080/0886022X.2023.2228419 (PMC10312025; doi:10.1080/0886022X.2023.2228419)
Supplement: Supplemental Material [file IRNF_A_2228419_SM8519.pdf]

Supplementary material_2

The significantly different metabolites in fecal samples.

Metabolites' name	Fold Change	P value	VIP	Up_Down
N-Acetylornithine	0.603115	0.00199	2.56183	down
5-Methoxytryptamine	0.437074	0.011711	2.139624	down
5'-Deoxy-5'-(Methylthio)Adenosine	0.492625	0.025697	1.888085	down
Hydroxyproline	0.572299	0.027898	1.849894	down
D-Cysteine	0.091779	0.029395	1.762232	down
Serotonin	0.471386	0.049351	1.64888	down
Capsaicin	2.372849	0.008808	2.249309	up
Homogentisic Acid	6.978546	0.014253	2.113807	up
Rosmarinic acid	6.280529	0.03179	1.911696	up
2-{[methyl(2,3,4,5,6-pentahydroxyhexyl)amino]methylidene}malononitrile	0.652878	0.000128	3.309987	down
L-(-)-Methionine	0.53506	0.00085	2.807898	down
2-[2-oxo-2-(2-pyridylamino)ethoxy]acetic acid	0.074167	0.001867	2.45513	down
Deoxylimonin	0.230178	0.002466	2.514336	down
1-(3-phenylpropanoyl)-4-piperidinecarboxylic acid	0.440057	0.003465	2.439251	down
3-Methoxybenzaldehyde	6.27761	0.004108	2.541787	up
9-(2,3-Dihydroxy-3-methylbutoxy)-7H-furo[3,2-g] chromen-7-one	1.719429	0.004369	2.364838	up
Dl-3-Hydroxynorvaline	0.465336	0.004944	2.36505	down
Inosine	0.641993	0.00589	2.335537	down
20β-Dihydroprednisone	2.779772	0.006515	2.600685	up
LPE 15:0	1.964138	0.008052	2.255074	up
gamma-Glutamylmethionine	0.662612	0.008893	2.231124	down
5-[(10Z)-14-(3,5-dihydroxyphenyl)tetradec-10-en-1-yl]benzene-1,3-diol	0.588838	0.009218	2.221174	down
2,4,5-Trimethoxybenzaldehyde	1.556729	0.009283	2.307643	up
Atropine	0.318597	0.009951	2.204978	down
3-(2,3-dihydro-1H-indol-1-yl)-2-[(2-furylmethyl)sulfonyl]acrylonitrile	2.518622	0.010059	2.230536	up
Pro-Leu	0.60281	0.013124	2.096397	down
(+/-)17(18)-EpETE methyl ester	1.919698	0.013404	2.247767	up
8-Hydroxyguanosine	0.410974	0.013731	2.048874	down
7-{[(2E)-3,7-dimethylocta-2,6-dien-1-yl]oxy}-2H-chromen-2-one	1.749647	0.01426	2.178833	up
Cytidine	0.523284	0.014574	2.053028	down
4-(4-chlorophenyl)-2-(3-pyridyl)-1,3-thiazole hydrobromide	0.558453	0.016055	2.032552	down
1-hydroxy-1-(4-methoxyphenyl)propan-2-yl	1.636944	0.016113	2.116203	up

Information Classification: General

4-methoxybenzoate				
N-α-L-Acetyl-arginine	0.514058	0.01638	2.091111	down
3-hydroxy-3,4-bis[(4-hydroxy-3-methoxyphenyl)methyl]oxolan-2-one	2.317879	0.017299	2.103118	up
Guvacoline	1.640427	0.017496	2.317566	up
3-(3-methylbut-2-en-1-yl)-3H-purin-6-amine	0.583562	0.018329	1.998801	down
SNK	0.493431	0.018342	1.982255	down
4-acetyl-4-(ethoxycarbonyl)heptanedioic acid	0.550971	0.019741	2.035491	down
(4-nitrophenyl)(2,3,4,5,6-pentamethylphenyl)methanone	0.60644	0.02021	2.036635	down
5-Hydroxyindole	2.963158	0.021007	2.46431	up
α-Aspartylphenylalanine	0.072979	0.021699	1.823146	down
Uracil 1-beta-D-arabinofuranoside	0.608019	0.023061	1.920643	down
4-morpholino-3-nitrobenzene-1-sulfonamide	0.490128	0.023069	1.951883	down
1-(5,7-dichloro-2,3,4,4a-tetrahydro-1H-xanthen-4-yl)pyrrolidine	0.556938	0.023462	1.954757	down
4-Methoxybenzaldehyde	5.442299	0.02358	1.930157	up
Lactobionic acid	0.489186	0.023798	1.931641	down
trans-2-Butene-1,4-dicarboxylic Acid	2.062036	0.024484	2.009904	up
Rhein	0.318038	0.02472	1.821908	down
methyl 4-oxo-4H-benzo[4,5]imidazo[2,1-b][1,3]thiazine-2-carboxylate	1.662005	0.025119	1.921455	up
LPE 22:5	1.630094	0.025236	1.93257	up
1-methyl-3,5-di(1-naphthylmethylidene)piperidin-4-one	3.097584	0.025476	1.986095	up
2-(acetylamino)-3-(1H-indol-3-yl)propanoic acid	0.332388	0.025853	1.850151	down

Information Classification: General
