# Supplementary material for: The gut microbiome and metabolome in kidney transplant recipients with normal and moderately decreased kidney function
Source: Ren Fail. 2023 Jun 29;45(1):2228419. doi: 10.1080/0886022X.2023.2228419 (PMC10312025; doi:10.1080/0886022X.2023.2228419)
Supplement: Supplemental Material [file IRNF_A_2228419_SM8514.pdf]

Supplementary material_1

Life style and simplified food frequency questionnaire

(The following content is only used for scientific research and is completely confidential.)

Name: ______________ Gender: __________ Age: __________ Phone number:_____________

Since after renal transplantation: ☐3 months ☐3-6 months ☐6-12 months ☐1-3 year ☐over 3 year

1. Medication:

1.1 Immunosuppressive treatment

☐ Cyclosporine (CsA, etc.) ☐ Tacrolimus

☐ Mofetil ☐ Glucocorticoid (prednisone, methylprednisolone, etc.) ☐

Other_______________

1.2 Other medication

1） Antibiotics ☐No ☐Yes, **:** _______________

2） Antihypertensive Agent ☐No ☐Yes, **:** _______________

3） Hypoglycemic drug ☐No ☐Yes, **:** _______________

4） Other ☐No ☐Yes, **:** _______________

2. Life style

Smoking habits: ☐ No ☐ Yes

Drinking habits: ☐ No ☐ Yes

Sport or exercise: ☐No ☐1-3 times per week ☐4-6 times per week ☐every day

The time of sport or exercise per week: _________hours

3. simplified food frequency

**How often do you eat the following types of foods?**<br>**（☑ for the circle, ✓ for the space on the right）**	every day	5-6 days a week	3-4 days a week	1-2 days a week	never
Staple food<br>○Mainly rice<br>○Mainly pasta<br>○Both rice and pasta					
Coarse grains (oats, corn, potatoes, mung beans, etc.)					
Fresh meat (pork, beef, chicken, etc.)					
Beans (tofu, peas, etc.)					
Processed meat (ham, bacon, luncheon meat, etc.)					
Fresh fish (shrimp, fish, crab, etc.)					
Milk (pure milk, yogurt, cheese, etc.)					
Fresh fruits (grapes, cantaloupe, apples, etc.)					
Vegetables (cabbage, pumpkin, etc.)					
Dietary supplements (vitamin A, calcium tablets, etc.)					
Sweets (cake, ice cream, chocolate, etc.)					
Non-alcoholic drinks (cola, soda, etc.)					
Alcoholic drinks (beer, etc.)					

Information Classification: General
